# Temporal variation in antibiotic environments slows down resistance evolution in pathogenic *Pseudomonas aeruginosa*

**DOI:** 10.1111/eva.12330

**Published:** 2015-10-07

**Authors:** Roderich Roemhild, Camilo Barbosa, Robert E Beardmore, Gunther Jansen, Hinrich Schulenburg

**Affiliations:** 1 Evolutionary Ecology and Genetics, University of KielKiel, Germany; 2 Biosciences, Geoffrey Pope Building, University of ExeterExeter, UK

**Keywords:** antibiotic cycling, antibiotic resistance, collateral sensitivity, experimental evolution, *Pseudomonas aeruginosa*, sequential therapy, temporal variation

## Abstract

Antibiotic resistance is a growing concern to public health. New treatment strategies may alleviate the situation by slowing down the evolution of resistance. Here, we evaluated sequential treatment protocols using two fully independent laboratory-controlled evolution experiments with the human pathogen *Pseudomonas aeruginosa* PA14 and two pairs of clinically relevant antibiotics (doripenem/ciprofloxacin and cefsulodin/gentamicin). Our results consistently show that the sequential application of two antibiotics decelerates resistance evolution relative to monotherapy. Sequential treatment enhanced population extinction although we applied antibiotics at sublethal dosage. In both experiments, we identified an order effect of the antibiotics used in the sequential protocol, leading to significant variation in the long-term efficacy of the tested protocols. These variations appear to be caused by asymmetric evolutionary constraints, whereby adaptation to one drug slowed down adaptation to the other drug, but not vice versa. An understanding of such asymmetric constraints may help future development of evolutionary robust treatments against infectious disease.

## Introduction

The extensive selective pressure of antibiotics in clinical, agricultural and, to some extent, natural environments continuously increases the pool of resistant bacterial mutants. The number of pathogens resistant to multiple antibiotics similarly grows rapidly (US Centers for Disease Control and Prevention [CDC], 2013), causing a surge of infections that resist treatment with standard broad-spectrum antibiotics (May 2014). One of the problematic examples is pan-resistant *Pseudomonas aeruginosa*, which evades treatment with any of the currently available antibiotics (Wang et al. 2006; Breidenstein et al. 2011). The antibiotic crisis is aggravated by a stagnant drug discovery pipeline (Hede 2014; May 2014) that in the last years has only yielded a handful of newly approved antibiotics for clinical use (e.g. CDC 2013; http://www.fda.gov database). Even novel methods seem unable to accelerate antibiotic discovery (Wright 2014). This leaves medicine almost empty-handed in the face of an ever-growing problem.

Even if drug discovery can pick up the pace, novel antibiotics are unlikely to solve the conundrum. New antimicrobials will eventually be rendered ineffective by the evolution of resistance, despite their initial success in treating infections. This is illustrated by antimicrobial peptides that were once advocated as invincible hurdles to bacterial evolution (Zasloff 2002). Yet bacteria readily evolved resistance to peptides (Perron et al. 2006; Habets and Brockhurst 2012; Lofton et al. 2013). More generally, these and related findings demonstrate the difficulty of managing an evolving organism using static, nonchanging countermeasures, such as application of a particular antibacterial compound. To employ the antibiotics already in our hands more efficiently, innovative treatment strategies therefore need to take evolution into account.

One promising treatment strategy is to alternate antibiotics with different cellular targets over the duration of a single therapeutical period. The resistance mechanisms selected by exposure to one of the antibiotics are likely to differ from those relevant for resistance against the other one, as long as general resistance mechanisms such as efflux pumps are not available or evolutionarily too costly. In this case, sequential drug application creates dynamic environments with temporally segregated selective pressures. As a consequence, only one resistance mechanism is beneficial at a time. If the resistance mechanism to at least one of the antibiotics is energetically or evolutionary costly (Lenski 1997), emergence of cross-resistance to both may be impeded (Perron et al. 2007). Moreover, the evolution of resistance to one antibiotic could sensitize bacteria against a second antibiotic (Szybalski and Bryson 1952). Such collateral sensitivity may additionally limit bacterial adaptation in sequential treatment regimes (Imamovic and Sommer 2013; Lazar et al. 2014; Oz et al. 2014). This idea was recently tested using evolution experiments with laboratory strains of *Escherichia coli* (Fuentes-Hernandez et al. 2015; Schenk et al. 2015) and *Staphylococcus aureus* (Kim et al. 2014), consistently demonstrating that antibiotic alternation decelerates adaptation rates.

In this study, we expand on the available work by specifically challenging a virulent strain of the human pathogen *P. aeruginosa* (PA14) with some of the clinically used antibiotics with different targets. PA14 was isolated from a human burn wound and is also virulent in many animals and plants (Rahme et al. 1995). Patients with cystic fibrosis often suffer from chronic *P. aeruginosa* lung infections and the bacteria rapidly evolve within the patients to the host environment, including the applied antibiotics (Damkiær et al. 2013; Marvig et al. 2015). Multidrug-resistant *P. aeruginosa* is an emerging pathogen threat recently highlighted by the CDC (CDC 2013). The first and main aim of our study was thus to assess to what extent the temporally variable application of antibiotics within one treatment regime (i.e. sequential treatment types) minimizes drug resistance evolution in the clinically relevant PA14 pathogen strain compared with the corresponding single-drug treatments (i.e. monotherapies). Our second aim was to test whether random rather than regular sequential therapy can further slow down resistance evolution, because randomly fluctuating sequences create less predictable environments. This idea is supported by experimental evolution of a RNA virus in alternative temperature regimes, where random changes across time limited and regular changes even facilitated adaptation to the environmental challenge (Alto et al. 2013). To address the two aims, we performed laboratory-controlled evolution experiments in which *P. aeruginosa* PA14 was treated with regular and random alternations of two antibiotics (regular or random sequential treatment types) or with the same antibiotics throughout the experiment (monotherapy treatment type). Most previous work on sequential therapy only compared regular antibiotic alternations to monotherapy (Kim et al. 2014; Perron et al. 2007; Schenk et al. 2015; but see Fuentes-Hernandez et al. 2015). These previous experiments and our study differ from the idea of ‘antibiotic cycling’ as tested in several clinical studies (e.g. Bennett et al. 2007; Martínez et al. 2006; Raymond et al. 2001; Sarraf-Yazdi et al. 2012; also see the meta-analysis of Abel zur Wiesch et al. 2014), in which antibiotics were not switched daily, within the treatment of one patient, but rather in monthly intervals, which is likely too long to constrain bacterial adaptation. An additional alternative to classical antibiotic cycling, which has not been explored in clinics, is the idea of alternating antibiotics in a noncyclic way by applying a random alternation schedule.

## Materials and methods

### Bacterial strains and media

We started all experiments from an isogenic population of the *P. aeruginosa* strain PA14. Bacterial populations were cultured in M9 minimal media supplemented with 0.2% glucose (w/v) and 0.1% casamino acids (w/v), according to a standard protocol (Hegreness et al. 2008). The bactericidal antibiotics cefsulodin (CEF), doripenem (DOR), gentamicin (GEN) and ciprofloxacin (CIP) – chosen for their clinical value in treating *P. aeruginosa* infections and because of their different targets – were added to this media at sub-MIC concentrations that inhibit growth of the common ancestors by 75% (IC_75_; see Fig. S2 in Supporting Information for dose–response curves). Antibiotic solutions were prepared according to the manufacturer’s instructions and stored at −20°C.

### Evolution experiment

We allowed populations of *P. aeruginosa* to evolve for a maximum of 100 generations in a serial transfer experiment consisting of 16 growth cycles (hereafter called ‘seasons’) of 12 h each. We performed two independent experiments of identical set-up with the antibiotic pairs CIP/DOR and GEN/CEF (experiments 1 and 2, respectively) and, in each case, a total of eleven treatment protocols that belonged to four treatment types: regular and random sequential treatments (2× four protocols), monotherapy (two protocols) and a no-drug control (see below). Each of the ten treatment protocols with an antibiotic was replicated eight times, while the control included 16 replicate populations, yielding a total of 96 replicate population per experiment (a total of 192 replicate populations for the two evolution experiments). During the experiment, we monitored population growth through continuous absorbance measurements (OD = optical density at 600 nm; GENios Spectra FLUOR plus, Tecan Austria GmbH, Grödig, Austria). Thus, we could track the evolutionary dynamics in real time. The data for the two experiments were analysed separately using the statistics software r (R Core Team, 2014).

We started the experiments by inoculating a microtitre plate with approximately 10^5^ cells/well from a liquid culture in mid-exponential growth phase. In an experimental season, the populations could grow to stationary phase (up to 10^8^ cells/well). At the end of each season, 1% of the culture volume was transferred to fresh media (equivalent to 100fold dilution) containing either the preceding or a new antibiotic at IC_75_ dosage (36 ng/mL DOR, 28 ng/mL CIP, 350 ng/mL CEF, 450 ng/mL GEN). The choice of IC_75_ dosage for our experiments represented a compromise to achieve two aims. On the one hand, bacterial cells still survive and can adapt. On the other hand, such dosage still exerts considerable selection pressure. Moreover, antibiotic dosage below the MIC is also clinically relevant, where the applied dosage is usually above MIC, but within the patient often lower due to, for example, incomplete tissue penetrance or drug degradation (Andersson and Hughes 2014). During our experiment, antibiotic concentrations were always kept constant.

Using two antibiotics, bacteria were challenged repeatedly by the same antibiotic (monotherapy treatment type) or by eight different successions of two antibiotics (the regular or random sequential treatment types). In four of the tested sequential treatment protocols, we switched antibiotics regularly, that is at every transfer or at every second transfer (treatment protocols 3–6). In four additional sequential protocols, the alternation of antibiotics followed a random temporal pattern (treatment protocols 7–10; see Online Supporting Information and Fig. S1 for details on how the random sequences were chosen). This set-up resulted in the following ten treatment protocols [antibiotics are indicated as A (DOR or CEF) and B (CIP or GEN)].

1. AAAAAAAAAAAAAAAA
2. BBBBBBBBBBBBBBBB
3. ABABABABABABABAB
4. BABABABABABABABA
5. AABBAABBAABBAABB
6. BBAABBAABBAABBAA
7. AABBABBBAABBBAAA
8. AABABBABBBBABAAA
9. BBABAAABBAAABABB
10. BAABAAABAAABBBBB

The effect of each treatment protocol was analysed with eight biological replicate populations. We also included 16 replicates of an evolving no-drug reference, yielding a total of 96 populations per evolution experiment. To avoid bias by gradients (Zimmermann et al. 2010), we systematically randomized treatments across a 96-well plate in a column-wise fashion.

### Analysis of resistance evolution

For each season, we evaluated the efficacy of antibiotic treatments by calculating the area-under-curve inhibition (AUC inhibition = 1 − AUC_drug_/AUC_no-drug_; R-package ‘MESS’; Ekstrom 2014) for each replicate population. This measure compares the complete growth curves of a drug-treated replicate population to a no-drug reference evolving in parallel. AUC inhibition represents a compound measure that depends on bacterial growth rate and lag phase, which are not directly taken into consideration by endpoint measurements (e.g. OD measurements at the end of a particular season). Moreover, AUC inhibition proved to yield an informative measure for tracking evolutionary dynamics across different drug treatments in previous work (Pena-Miller et al. 2013; Fuentes-Hernandez et al. 2015). High values of AUC inhibition denote strong growth inhibition by the drugs, whilst low values correspond to low inhibition. A decrease in AUC inhibition thus marks the evolution of resistance.

Statistical analysis of treatment efficacy across an evolution experiment was based on a mixed linear model (R-package ‘nlme’; Pinheiro et al. 2013) with AUC inhibition as response variable, treatment protocol and season as fixed factors and replicate population (=selection line) within a particular treatment as a nested random factor. The *P*-values were obtained from *post hoc* tests and corrected for multiple testing using the false discovery rate (R-package ‘multcomp’ and adjustment of *P*-values by ‘fdr’; Hothorn et al. 2008). We further evaluated, whether antibiotic alternation decelerates adaptation to the individual components of the antibiotic pairs by collating the measurements of AUC inhibition for the eight (out of sixteen) seasons in the sequential treatment, during which populations were exposed to only one specific antibiotic (e.g. only the A seasons in a ABABABABABAB treatment). For this measurement, we thus ignored the other eight seasons, during which the populations were exposed to antibiotic B. These values were compared to the first eight seasons of the respective monotherapies (in this example, of A) using a mixed linear model as described above (AUC inhibition as response variable, time and treatment protocol as fixed factors and replicate populations as a nested random factor).

As an additional measure for bacterial adaptation, we calculated cumulative OD for the different treatment protocols by summing the endpoint OD measurement of each season across all seasons. This measure specifically summarizes the total bacterial yield during the sixteen seasons of antibiotic treatment, and thus, it is related to above AUC inhibition measures, yet depicts a different aspect of the evolutionary response of the bacterial populations. We compared means of cumulative OD between treatment protocols using nonparametric Welsh *t*-tests and accounted for multiple testing by adjusting *P*-values using the false discovery rate (fdr).

### Analysis of sequential treatment dynamics

We performed frequency tests to assess whether switch to one of the antibiotics generally led to a significant increase in inhibition in the sequential treatment protocols. For each replicate population, we counted the number of antibiotic switches during which inhibition increased relative to the preceding value. If the average was higher than 50% of the total number of switches for a particular replicate population, the score of the respective replicate was set to 1. Otherwise, it was set to 0, indicating either no change or a decrease in inhibition due to the antibiotic switch. Next, the frequency of 0s and 1s were counted for each individual treatment protocol (which all included eight replicate populations). If the majority were 1s, the treatment protocol received an overall score of 1, otherwise 0. This procedure was done separately for the switch from A → B and B → A. By combining the information across replicate populations for each treatment protocol, we obtained a conservative measure for the effect of a drug switch. For each evolution experiment, we thus obtained 16 count values (two for each of the eight sequential treatment protocols), which were evaluated using a Fisher’s exact test. The thus obtained *P*-value indicates the significance of zig-zag growth dynamics due to drug order.

### Separate analysis of collateral sensitivity and resistance

After the evolution experiments, we experimentally tested for the evolution of collateral sensitivity and resistance. We focused on evolved material from seasons 6 and 7 from the sequential treatment protocol 4, which showed a particularly pronounced zig-zag pattern (see Results). Seasons 6 and 7 correspond to the valley and peak of the zig-zag curve. For feasibility, we randomly chose three of the originally eight replicate populations of these treatment protocols for further analysis. For these replicate populations, we revived evolved bacterial lines from frozen glycerol stocks by plating them out on agar plates containing the particular antibiotic which was experienced by the line prior to freezing (M9 agar with IC_75_ of the drug). After 24 h of incubation, we randomly sampled five individual colonies from each replicate population and season and cultured them as in the evolution experiment (i.e. with the same antibiotic). As references, we grew five colonies from the ancestral population in liquid culture without antibiotics. Next, we transferred 1% of the liquid cultures to five concentrations of the same antibiotic and separately to five concentrations of the other antibiotic of the evolution experiment. The cultures of the ancestor were transferred to all four antibiotics and all concentrations. We measured optical density after 12 h of incubation and calculated the area under the dose–response curve. Note that this measure differs from AUC inhibition, which is instead derived from the area under the time-dependent response curve. For statistical analysis, we compared corresponding values for the same antibiotic between evolved clones and ancestral populations using a mixed linear model with colonies nested in replicate populations.

## Results

**Figure 1:**
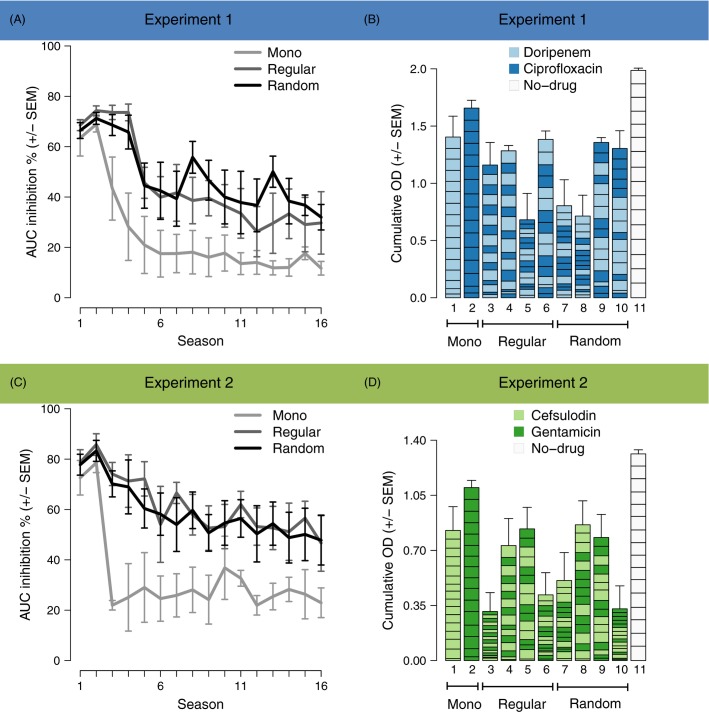
Temporal variation in drug environments decelerates adaptation. (A), (C) Antibiotic inhibition (AUC inhibition, see Methods) for the three treatment types in experiments 1 and 2, respectively. The evolutionary dynamics in regular and random sequential protocols differ significantly from those of the monotherapy but not from each other. Error bars represent standard error of the mean (SEM) of treatment groups: *n* = 2 for Mono and *n* = 4 for Regular and Random. (B), (D) Total amount of bacterial growth during antibiotic treatment (cumulative OD) in experiments 1 and 2, respectively. Cumulative OD is significantly lower in temporally variable than in constant environments. Variation among protocols is high with relation to drug order. Error bars represent SEM of replicate populations: *n* = 8 for treatments 1–10; *n* = 16 for treatment 11.

In populations treated with a single antibiotic, inhibition by the antibiotics decreased within the first six transfers and remained relatively constant thereafter (Fig. 1A,C). The decline of inhibition was significantly slower in populations challenged by regular or random sequences of the two antibiotics (Fig. 1A,C; Table1). Sequential challenge thus prevented an escape from treatment by rapid adaptation. These results indicate that temporal heterogeneity in drug environments prolongs treatment efficacy. Regular and random sequential treatment protocols equally suppressed bacterial growth over time (Fig. 1A,C; Table1); the success of sequential therapy appears to be independent of the temporal regularity of drug exchange. These findings were confirmed if results were summarized as cumulative OD. This measure approximates the total bacterial growth during treatment, and therefore provides a separate measure of treatment efficacy across the entire duration of the experiment. Here, sequential treatment protocols led to significantly lower cumulative OD than monotherapies (Fig. 1B,D; Table S3).

**Table 1:** Significance of AUC inhibition over time from mixed linear models

	Experiment 1	Experiment 2
Mono versus Regular	0.0029^*^^*^	<0.001^*^^*^^*^
Mono versus Random	0.0005^*^^*^^*^	0.0021^*^^*^
Regular versus Random	0.3871	0.5934

The table shows *P*-values adjusted by the false discovery rate (^*^^*^P < 0.01, ^*^^*^^*^P < 0.001). The models consider replicate populations as random factor, which was significant in the likelihood ratio test (Experiment 1: *P* < 0.0001, LR = 705.86; Experiment 2: *P* < 0.0001, LR = 940.31).

To explain why sequential treatments outperform monotherapies, we analysed potential factors that may underlie the success of some protocols over others. Particularly, we investigated whether the outcome of a sequential protocol could be predicted by (i) adaptation over the length of the protocol to only one of the two antibiotics in the sequence (as if one antibiotic was exerting the dominant selective force, whereas adaptation to the other played no role), (ii) the starting drug of the sequence, that is, the drug used during the first season alone, or (iii) the cost of evolving resistance to the first-season drug. We also asked whether the observed zig-zag evolutionary dynamics could be explained by (iv) overall variation in the order of antibiotics across the entire sequence or (v) collateral sensitivities between the two antibiotics. Finally, we determined (vi) extinction frequencies of the different types of protocols.

**Figure 2:**
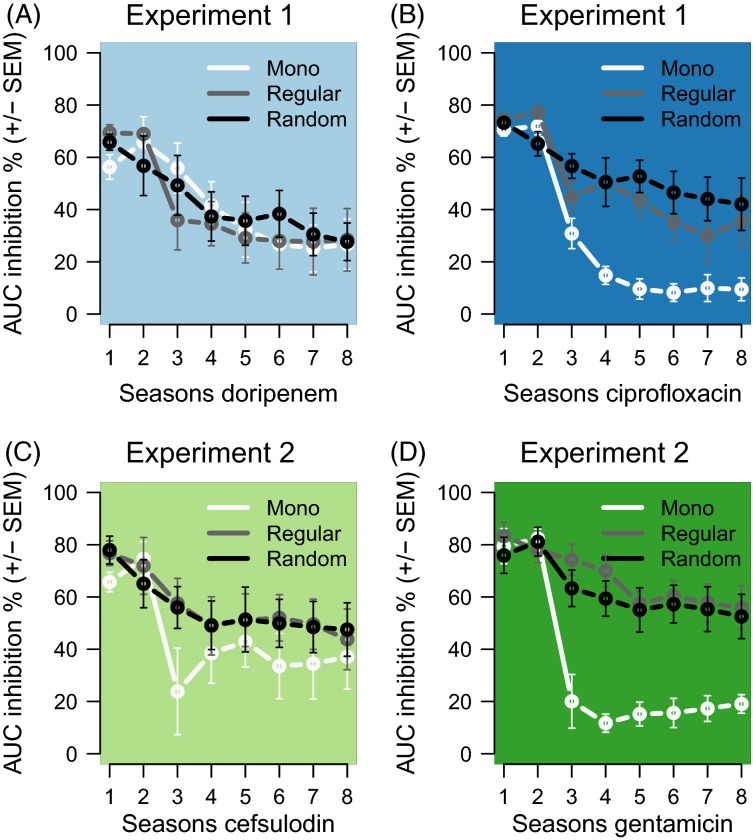
Antibiotic alternation decreases adaptation rates across seasons of exposure to the same antibiotic. The graphs show the chronological collation of AUC inhibition values during exposure to always the same antibiotic, excluding AUC inhibition values during intermittent exposures to a different antibiotic in the alternating protocols. (A) Doripenem, (B) Ciprofloxacin, (C) Cefsulodin and (D) Gentamicin. On the timescales of the non-β-lactam antibiotics (panels B and D), AUC inhibition decreases more slowly in the alternating treatments compared with the corresponding monotherapies. This demonstrates that intermittent exposure of populations to the β-lactam antibiotics decelerates adaptation. The lines show mean AUC inhibition for the corresponding monotherapy and the regular and random alternations (*n* = 1 for Mono and *n* = 4 for Regular and Random) always across the eight seasons, during which the bacteria were exposed to these antibiotics.

Our first analysis assessed whether decelerated adaptation was caused by the sequential treatment protocol itself or merely by the number of seasons during which a particular replicate population was exposed to a certain antibiotic, irrespective of intermittent seasons with the other antibiotic. For this test, we plotted the chronologically obtained AUC inhibition values for the seasons during which only one of the antibiotics was used, thus excluding all AUC inhibition values obtained for intermittent seasons with the other antibiotic. The results are shown in Fig. 2. For the collated DOR seasons (i.e. the DOR time scale), inhibition decreased with identical slope in the sequential and monotherapy treatment types (Fig. 2A). Accordingly, a disruption of a DOR-monotherapy by CIP did not significantly affect the rate of adaptation (Table S1 for statistics). In contrast, for CIP, inhibition decreased significantly faster in monotherapies than in regular or random sequential treatment types (Fig. 2B; Table S1). Thus, interruption of a CIP monotherapy by DOR seems to have decelerated adaptation to CIP. We found a similar asymmetric pattern in Experiment 2. Here, inhibition decreased significantly faster in monotherapies than during the GEN seasons in the sequential treatment types (Fig. 2C; Table S1) but not so during CEF seasons (Fig. 2C; Table S1). Hence, a disruption of a GEN monotherapy by CEF decelerated adaptation. We conclude that it is the alternation of drugs that caused a decreased rate of adaptation to antibiotic treatment.

Second, we asked which specific aspect of the sequential treatment protocols accounts for decelerated adaptation? Interestingly, cumulative OD varied strongly among regular sequential protocols (Fig. 1B,D), indicating that the order within a given pair of antibiotics determined treatment potency. A possible order effect may be caused by the first antibiotic applied. For evolution Experiment 2, the regular sequential treatment protocol 5 that started with CEF led to radically higher cumulative OD than the mirrored sequential treatment protocol 6 that started with GEN (Fig. 1D; bars 5 and 6; Table S3). However, a similar significant difference between treatment protocols 5 and 6 could not be observed for evolution Experiment 1. In contrast, for Experiment 1, it is the overall treatment outcome (across the various protocols) that depended significantly on the antibiotic applied in the first season (i.e. the *β*-lactam DOR in the first season across the various treatments led to lower accumulative OD; Fig. 1B; Table S3), while the same overall effect was insignificant for Experiment 2 (Table S3). We conclude that the first-season drug cannot fully explain the observed variation in treatment outcome among sequential protocols.

Third, to test in how far a possible evolutionary cost of resistance against the antibiotic encountered in the first season may account for the observed cumulative variations, we analysed growth parameters of the monotherapy-treated populations in a drug-free season succeeding the evolution experiments. We found no significant differences in exponential growth rate between populations adapted to *β*-lactam antibiotics (DOR, CEF) and CIP- or GEN-adapted populations (Fig. S4). Thus, we refute asymmetric costs of resistance as an explanation for the overall variation in treatment efficacy.

**Figure 3:**
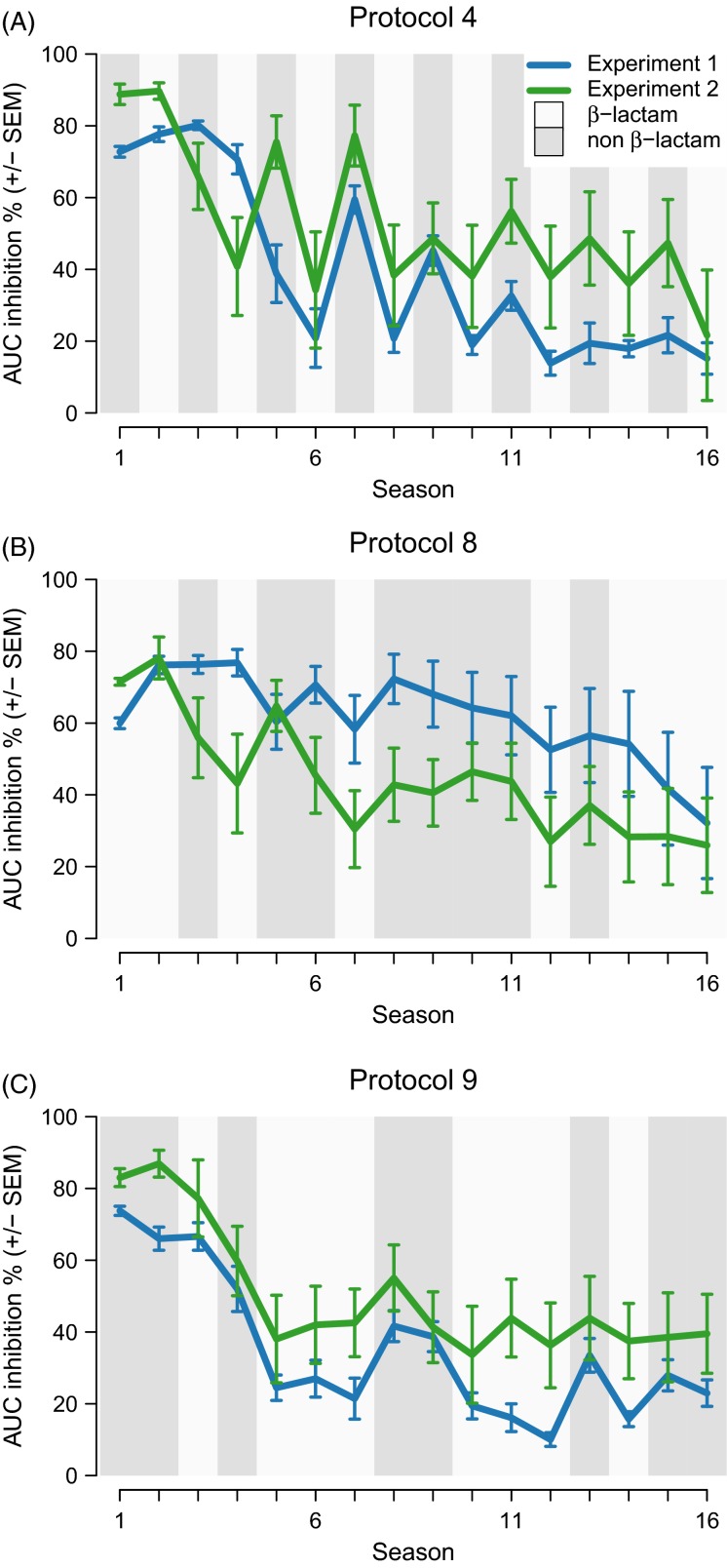
Evolutionary zig-zag dynamics. (A), (B), (C) Antibiotic inhibition for three exemplary sequential protocols in the two experiments. Antibiotic presence corresponds to the background shading. Inhibition oscillates after the first drop in inhibition. The peaks of inhibition mostly correspond to the non-β-lactam, whilst valleys mostly coincide with the β-lactam. Error bars represent standard error of the mean (SEM) of replicate populations (*n* = 8).

Fourth, we investigated the general role of drug order across the entire sequential protocol in determining antibiotic adaptation. Such a role is supported by the exact growth dynamics in the sequential treatment protocols. A switch from the *β*-lactam antibiotic DOR to the non-*β*-lactam antibiotic CIP in Experiment 1 was significantly associated with an increase in inhibition (Fisher exact test, *odds ratio *= Inf, *P *= 0.0035). Conversely, the opposite switch from CIP to DOR decreased inhibition, thereby forming zig-zag patterns across time. Figure3 illustrates these growth dynamics for three sequential treatment protocols (Figs S5–S6 for the full data set). We found a similar pattern in Experiment 2: inhibition increased when antibiotics switched from the *β*-lactam to the non-*β*-lactam antibiotic – from CEF to GEN – and decreased when antibiotics were changed reversely (Fisher exact test, *odds ratio *= 16.20, *P *= 0.0203). These dynamics suggest that adaptation to one antibiotic affects the ability to adapt to another one.

**Figure 4:**
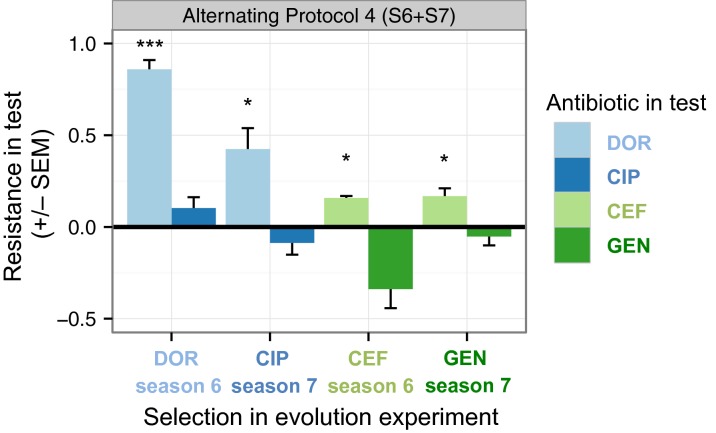
Evolution of collateral effects. Collateral profile of evolved populations from alternating treatment 4 that were isolated from seasons 6 and 7, which correspond to pronounced zig-zag patterns in the growth dynamics during experimental evolution (Fig. 3A). The collateral profile was calculated from the area under the dose-dependent response curve (AUC) with the equation: resistance in test = (mean AUC_evolved_ − mean AUC_ancestor_)/mean AUC_ancestor_. Positive values denote resistance and negative values denote collateral sensitivity. This measure differs from AUC inhibition which is instead derived from the area under the time-dependent growth curve. Each bar is based on 15 dose–response curves (3 replicate populations × 5 independent clones from these). Error bars represent standard error of the mean (SEM) of the populations (*n* = 3). The asterisks indicate significance of fdr-adjusted *P*-values (*P* < 0.05*, *P* < 0.01**, *P* < 0.01***). AUC: area under the dose–response curve, DOR: doripenem, CIP: ciprofloxacin, CEF: cefsulodin, GEN: gentamicin.

Fifth, a more specific explanation for the observed zig-zag pattern could be provided by the evolution of collateral sensitivities. To test this aspect, we measured the dose–response curves against both antibiotics of a particular pair. We focused the analysis on independent clones isolated from three randomly chosen replicate populations of seasons 6 and 7 of the antibiotic alternation treatment protocol 4, which showed a pronounced zig-zag pattern (Fig. 3A). In both evolution experiments, the clones isolated from season 6 of treatment 4 (presence of DOR in Experiment 1 or CEF in Experiment 2) were resistant to the antibiotic present in that particular season (Fig. 4, Experiment 1: *P* < 0.001, Experiment 2: *P* = 0.030, Table S2) but not resistant to the other antibiotic of the pair. Instead, in Experiment 2, the resistance to CEF was associated with collateral sensitivity to GEN, although the effect was insignificant (Fig. 4, *P* = 0.069, Table S2). Clones sampled from season 7 of the same experiment (presence of GEN), no longer showed this signature of collateral sensitivity, but were still resistant to CEF (Fig. 4, *P* = 0.030, Table S2). This observation may suggest persistence of at least some bacteria that previously evolved resistance to CEF. In clones from Experiment 1 season 6, we saw no evidence for the evolution of collateral sensitivity. In this case, resistance to DOR decreased in season 7 but stayed significant, indicating weak counterselection of the DOR-resistant bacteria by CIP. We did not find significant cross-resistances in either evolution experiment. Taken together, our results for the tested sequential treatments provide only weak indications of asymmetric collateral sensitivity, which is therefore unlikely to be the sole explanation for the observed evolutionary dynamics.

**Figure 5:**
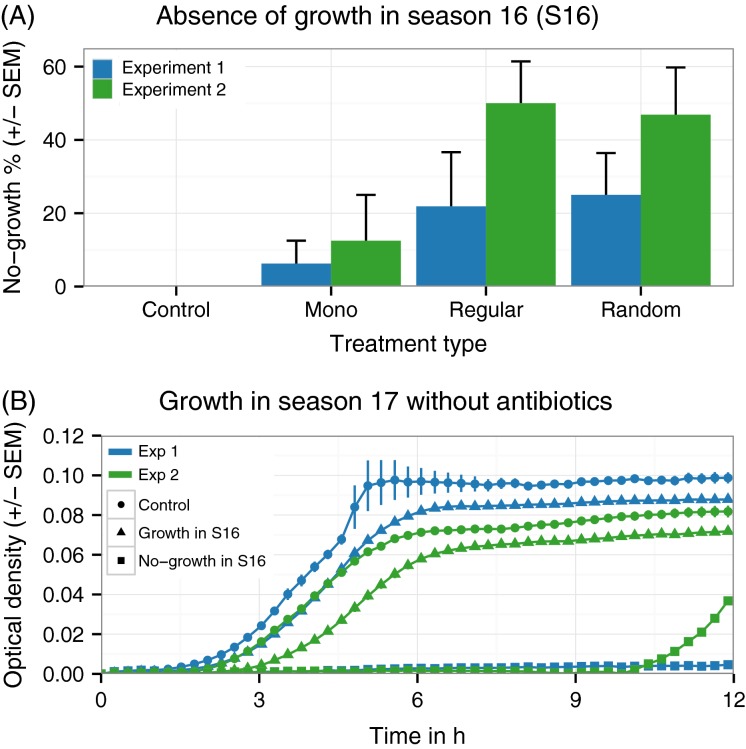
Antibiotic alternation enhances extinction of bacterial populations. (A) In both experiments, certain populations show no growth in season 16, although antibiotics were applied at sublethal concentrations. This effect occurred more frequently when two antibiotics alternated (Regular, Random) than in monotherapy (Mono). Error bars represent standard error of the mean (SEM) of treatment groups (*n* = 2 for Mono; *n* = 4 each for Regular and Random). (B) Growth curves in media without antibiotics in season 17. The populations are grouped according to their ability to grow in the preceding season 16, as indicated by the different symbols. The lines are the mean of all populations that fall into the three categories, including control (*n* = 16 in exp. 1; *n* = 16 in exp. 2), growth (*n* = 64 in exp. 1; *n* = 47 in exp. 2), and no-growth (*n* = 16 in exp. 1; *n* = 33 in exp. 2). In Experiment 1, populations that showed no growth in season 16 also showed no growth in season 17. In Experiment 2, populations that showed no growth in season 16 restarted growth after a long lag-phase.

Finally, by the end of the experiment, some treatment protocols fully suppressed growth of certain replicate populations although we always applied antibiotics at sublethal concentrations (dosage initially allowed for 25% growth; Fig. 5A). Sublethal dosage thus drove replicate populations below the detection limit of the plate reader. In Experiment 1, these suppressed populations were eradicated – they did not recover during the additional season without antibiotics (season 17; Fig. 5B). Population growth suppression occurred more often for the sequential treatment protocols than monotherapies, although this trend was not significant (Fig. 5A; Fisher’s exact test, *P* = 0.1719, *odds ratio *= 0.2208). In Experiment 2, suppressed replicate populations were significantly more frequent in the sequential treatment protocols (Fig. 5A; Fisher’s exact test, *P* = 0.0104, *odds ratio *= 0.1552), yet they all recovered in antibiotic-free media, after a lag-phase of approximately 10 h (Fig. 5B). The slow transition to steady-state growth suggests that in this case bacteria survived across time through a persister-phenotype. We conclude that sequential treatment protocols have the potential to eliminate bacteria at sublethal drug concentrations.

## Discussion

We experimentally evolved *P. aeruginosa* in temporally constant or variable drug environments. For two pairs of clinically relevant anti-pseudomonal antibiotics (doripenem/ciprofloxacin and gentamicin/cefsulodin), our study demonstrates that the alternation of antibiotics significantly minimizes adaptation rates (Figs1 and 2) and leads to more population extinctions (Fig. 5). These effects appear to depend on the exact sequential order, in which the two antibiotics of a particular pair are applied across time. This is most clearly seen for the regularly changing sequential treatment protocols (Figs1B,D and 3). In contrast, irregularity of change (compared to regular alterations) only seems to have a minor effect on resistance evolution. Our study provides one of the few case examples for which a defined treatment protocol with available antibiotics consistently reduced the likelihood of resistance evolution in a bacterial pathogen.

For *P. aeruginosa*, our finding of a general drug order effect on overall treatment efficacy is supported by a previous evolution experiment, in which a different strain of this pathogen (PAO1) was exposed to regular one-season alternations of rifampicin and streptomycin (Perron et al. 2007). In this previous experiment, cross-resistance developed later when treatment started with the antibiotic with the higher cost of resistance (rifampicin; Perron et al. 2007). Cross-resistance developed earlier and showed zig-zag growth dynamics when treatment started with the antibiotic with lower cost of resistance (streptomycin; Perron et al. 2007). Our results are consistent with the zig-zag growth patterns and the overall effect of antibiotic switches on growth. Yet, for the antibiotics of this work, we could not find significantly different costs of resistance (Fig. S4) which may explain why we cannot consistently replicate the observation that the antibiotic encountered first determines treatment success (Fig. 1B,D). Consequently, we cannot explain the observed drug order effect by costs of resistance.

A general drug order effect may alternatively result from the bacteria’s inability to achieve cross-resistance to both drugs in one step, for example through a single molecular mechanism, such as a change or amplification of the multidrug efflux system *mexAB-oprM* operon (Poole 2001). Otherwise we would have expected inhibition to change smoothly across time. Yet, we observed zig-zag patterns (Fig. 3), suggesting cross-resistance to arise sequentially by distinct mechanisms and with nonidentical efficiencies (i.e. due to an asymmetrical capacity for resistance evolution). This alternative is in accord with the currently known diversity of resistance mechanisms in this pathogen. *P. aeruginosa* mostly achieves resistance against the antibiotics used by us through individual mechanisms, which rarely produce cross-resistance (reviewed by Poole 2011). Hence, temporally heterogeneous drug environments may alternately select for different rather than a common resistance mechanism. The resistance mechanisms may then evolve with different likelihoods. The results obtained for the monotherapy protocols suggest that resistance can evolve faster for the non-*β*-lactams CIP and GEN, because their overall yield is higher and their AUC inhibition reaches lower values faster than the corresponding values for the *β*-lactam monotherapies (the two left bars in Fig. 1B,D; white lines in Fig. 2). In contrast, however, the zig-zag patterns of the alternating treatments always show the lowest AUC inhibition values for the *β*-lactams and the peaks for the non-*β*-lactam (Fig. 3). Therefore, an asymmetrical capacity to adapt to the antibiotics of a pair does not seem to explain the zig-zag patterns consistently observed in our evolution experiments.

Resistance mutations that adapt against one antibiotic may also amplify the potency of the other antibiotic. Such collateral sensitivities were recently described for various pairs of antibiotics in *E. coli* (Imamovic and Sommer 2013; Lazar et al. 2014; Munck et al. 2014; Oz et al. 2014) and *S. aureus* (Kim et al. 2014) and indicated in both bacteria to account for reduced resistance evolution during sequential drug protocols (Kim et al. 2014; Fuentes-Hernandez et al. 2015). For a pair of antibiotics, A and B, collateral sensitivity can take two main forms, being either reciprocal or asymmetric. In the case of reciprocal collateral sensitivity, adaptation to either antibiotic sensitizes against the other antibiotic of the pair. In the case of asymmetric collateral sensitivity, adaptation to A sensitizes against B, but adaptation to B increases resistance to A or has no effect. Such asymmetrical collateral effects were recently linked with bacterial population extinction (Fuentes-Hernandez et al. 2015) and may thus also account for our observations of full growth suppression in Experiment 2 and population eradication in Experiment 1 (Fig. 5). The observed zig-zag growth patterns are similarly consistent with an evolution of asymmetric collateral sensitivities. In particular, the evolution of collateral sensitivity by adaptation to the *β*-lactam antibiotic could explain why inhibition usually spiked upon switches to the non-*β*-lactam. Conversely, exposure to the non-*β*-lactam was followed by decreased inhibition in a *β*-lactam environment, possibly indicating evolution of collateral resistance. However, our separate analysis of evolved lines from intermediate seasons of treatment protocol 4 provides only weak support for the importance of asymmetric collateral effects (Fig. 4).

The observed zig-zag pattern thus requires an alternative explanation. Possible factors include the differential speed, with which resistant mutants to either of the antibiotics may be able to spread within the evolving populations. In particular, it is possible that *β*-lactam-resistant mutants can spread faster than the mutants with resistance to the non-*β*-lactam, leading to high prevalence of the *β*-lactam-resistant variants under *β*-lactam conditions (and thus low AUC inhibition, Fig. 3), whereas the non-*β*-lactam-resistant mutants would only reach intermediate frequencies under the non-*β*-lactam conditions (resulting in higher values of AUC inhibition, Fig. 3). Such a scenario could then produce the observed zig-zag dynamics during the evolution experiment and also account for the missing evidence of asymmetrical collateral sensitivity in our separate analysis. As an alternative, it is similarly conceivable that more than one locus cause some of the observed resistances and associated collateral effects, potentially resulting in epistatic interactions between loci and thus also complicating the analysis of such effects. Yet another alternative is that any of the above processes act jointly to cause the zig-zag dynamics and would thus only produce weak effects if assessed individually. Future dissection of these alternatives may benefit from an identification of the involved loci and their functional genetic analysis and/or an analysis of resistance patterns of individual cells within the evolving populations.

Even though the exact underlying process is still unclear, our data clearly demonstrate that adaptation to the antibiotics of a pair is shaped by asymmetric evolutionary constraints. Interestingly, such asymmetric effects are thought to be a common outcome of adaptation to local ecological conditions. Constant local conditions favour the evolution of ecological specialists capable of utilizing the available resources more efficiently than their ancestor (Kassen 2002). Their higher fitness in this particular environment, however, may be coupled to costs revealed in another one. Such trade-offs are then often asymmetric (Kassen 2002), as for example demonstrated in a long-term evolution experiment cultivating *E. coli* at hot and cold niche extremes (Mongold et al. 1996). Cold-adapted lines tolerated hot temperatures less than their ancestor, representing a fitness cost of adaptation (Mongold et al. 1996). The hot-adapted lines, however, were equally fit at the cold temperature; that is, their genetic changes were cost-free in this context (Mongold et al. 1996).

In temporally heterogeneous environments, however, reports of such asymmetric evolutionary constraints are rare. Rather, temporal environmental variation most often selects for cost-free generalists that are equally or superiorly fit across the range of environmental variation (Kassen 2002; Buckling et al. 2007; Duncan et al. 2011; Ketola et al. 2013). Environmental contrasts often involve specific genetic changes, such that, when substrates are mixed, generalists may evolve slowly (Zhong et al. 2004) or are actively selected against (Jasmin and Kassen 2007) due to antagonistic pleiotropy. Like sugars, antibiotics target very specific structures, thereby exerting disparate selective pressures. In the temporally heterogeneous environments created by our sequential drug protocols, we found faster evolution of specialists (resistant to one antibiotic as selected in monotherapies) than generalists (lines resistant to both antibiotics). This is consistent with increased divergence of *E. coli* lines evolved in alternations of lactose and glucose (Cooper and Lenski 2010). Asymmetric evolutionary constraints may thus impede the evolution of generalists, both in certain antibiotic and antibiotic-free environments.

In conclusion, we here provide evidence that temporal variation in drug environments minimizes adaptation rates relative to constant single-drug environments. Sequential antibiotic treatment significantly decelerated resistance evolution independent of the temporal regularity by which drugs were switched. Asymmetric evolutionary constraints account for reduced adaptation in the changing environments. Their future analysis, including dissection of the underlying genetic mechanisms, may help to develop evolutionary robust treatments of bacterial infections.

## Data archiving statement

Data available from the Dryad Digital Repository: http://dx.doi.org/10.5061/dryad.c867b.

## Supporting Information

**Appendix S1.** To test whether the irregularity of a drug protocol impacts the evolution of resistance, the level of randomness needs to be precisely defined. **Table S1.** Significance of AUC inhibition across separate timescales for each antibiotic. **Table S2.** Summary of statistics for the assessment of collateral sensitivity. **Table S3.** Summary of statistics for comparisons of cumulative OD.

**Figure S1.** Sampling of sequential protocols with high randomness scores.

**Figure S2.** Dose-response curves.

**Figure S3.** Time-growth curves of the first season in experiment 1.

**Figure S4.** Fitness costs of evolving antibiotic resistance.

**Figure S5.** Evolutionary zig-zag dynamics for all treatments of experiment 1.

**Figure S6.** Evolutionary zig-zag dynamics for all treatments of experiment 2.
