# Supplementary material for: Temporal variation in antibiotic environments slows down resistance evolution in pathogenic Pseudomonas aeruginosa
Source: Evol Appl. 2015 Oct 7;8(10):945–55. doi: 10.1111/eva.12330 (PMC4662347; doi:10.1111/eva.12330)
Supplement: Supplementary file 1 [file eva0008-0945-sd1.pdf]

Temporal variation in antibiotic environments slows down resistance evolution in pathogenic *Pseudomonas aeruginosa*.

Online Supporting Information

Appendix S1 1
Figure S1. Sampling of sequential protocols with high randomness scores. 3
Figure S2. Dose-response curves. 4
Figure S3. Time-growth curves of the first season in experiment 1. 5
Figure S4. Fitness costs of evolving antibiotic resistance. 6
Figure S5. Evolutionary zig-zag dynamics for all treatments of experiment 1. 7
Figure S6. Evolutionary zig-zag dynamics for all treatments of experiment 2. 8
Table S1. Significance of AUC inhibition across separate timescales for each antibiotic. 9
Table S2. Summary of statistics for the test for collateral sensitivity. 10
Table S3. Summary of statistics for comparisons of cumulative OD. 11
Supporting references 12

Appendix S1

To test whether the irregularity of a drug protocol impacts the evolution of resistance, the level of randomness needs to be precisely defined. We defined a randomness measure that is based on Shannon entropy (H; Shannon 1948). Shannon entropy quantifies the information content of a sequence that was attained by sampling from a defined alphabet:

(1) Shannon entropy

$$H = -\sum_{i=1}^{n} p_i \log_2 p_i$$

p_i relative proportion of a drug in a sequence

n number of drugs

(2.1) Shannon entropy for a sequence with two drugs A and B

$$H = -(p_A \log_2 p_A + p_B \log_2 p_B)$$

(2.2) Modified Shannon entropy (inverted sign)

$$H = -(p_A \log_2 p_A - p_B \log_2 p_B)$$

H only depends on the relative proportion of the drugs, so that they can be reordered without changing the result, *e.g.* H(ABBAB) = H(AABBB). To include temporal order into the randomness measure, H was calculated using a sliding window approach. The overall randomness measure R of the entire sequence is the mean H over all windows (equation 3). We modified H to obtain normally distributed values of R (Fig. S1A) by calculating the difference instead of the sum in each window (equations (2.1) and (2.2)).

(3) Randomness measure

$$R = \frac{1}{m} \sum_{j=1}^{m} H_j$$

m number of windows

All possible sequences with equal proportions of two drugs and a length of 16 were generated using a Python script. To avoid extended runs (period in which the drug is not changed), the constraint was added that drugs must be changed at least 5 times. We calculated R for each sequence (sliding window of size 5) and then randomly sampled four sequences from the top 30% tail of the distribution (Fig. S1B). The thus obtained sequence protocols are designated "random" in the evolution experiments.

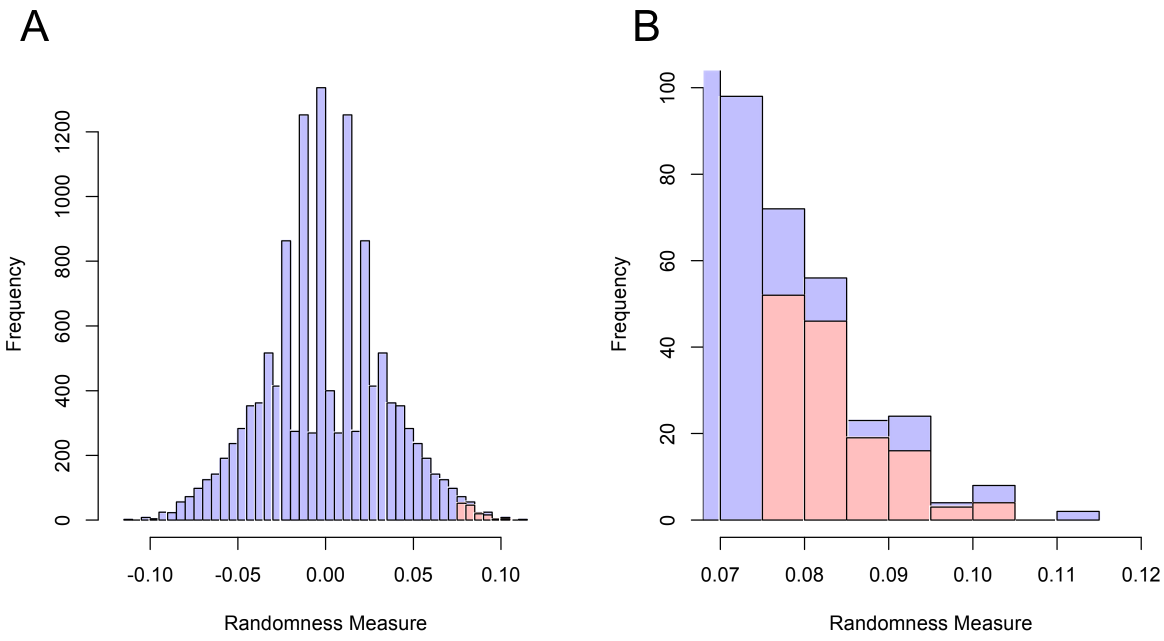

Figure S1. Sampling of sequential protocols with high randomness scores.

(A) Distribution of the randomness measure R of all possible sequences with equal proportions of drugs A and B. The quantile, from which protocols were sampled, is highlighted in red. (B) Magnification of the positive tail. The most random sequences had extended runs and were therefore excluded.

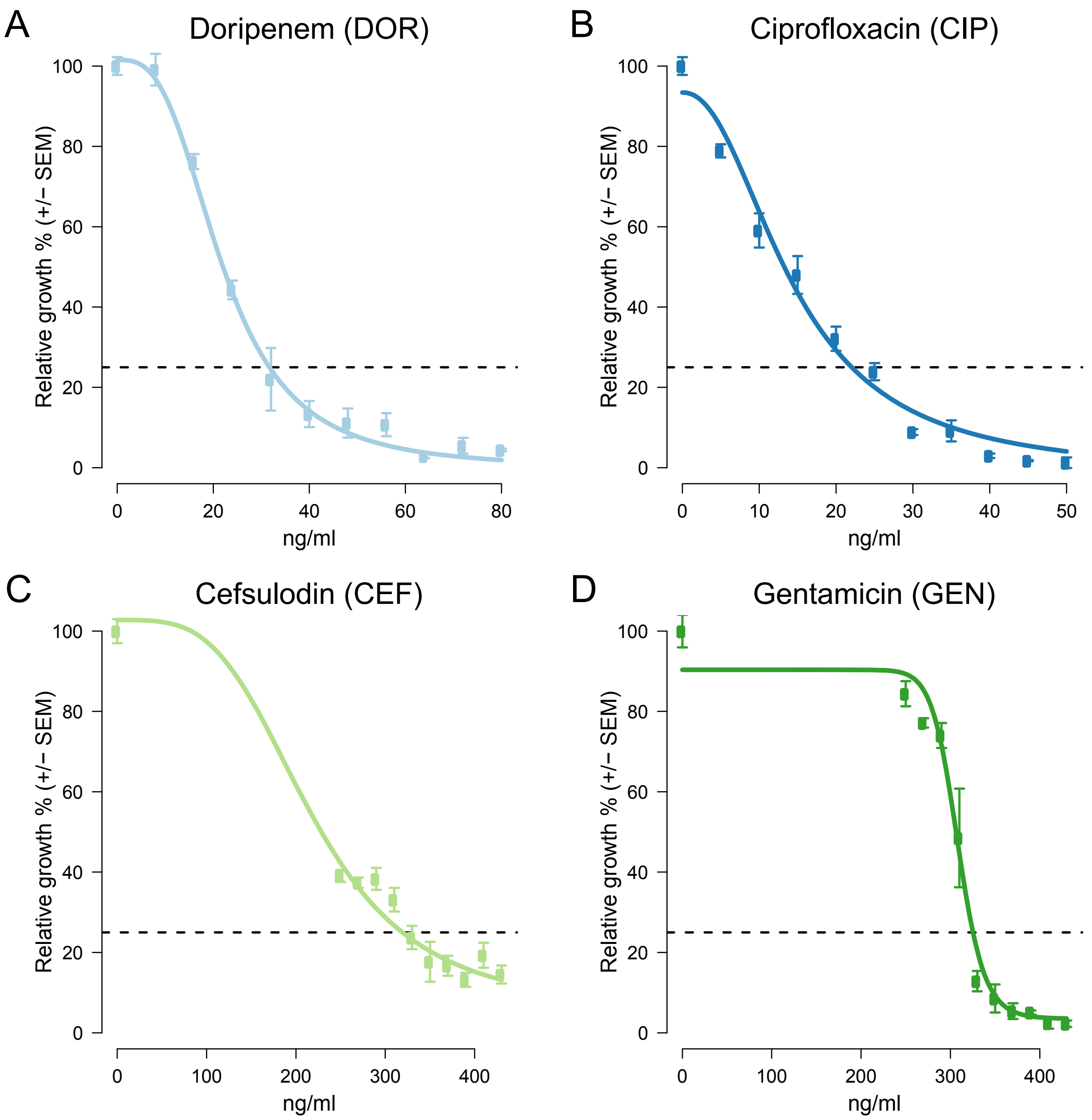

Figure S2. Dose-response curves.

(A) Ciprofloxacin, (B) doripenem, (C) cefsulodin and (D) gentamicin. Points indicate optical density relative to a no-drug reference after 12 h of incubation. Solid lines show a logistic model describing the dose-response function (R-package 'drc'; Ritz and Streibig 2005). The dashed horizontal lines indicate 75% inhibition of growth.

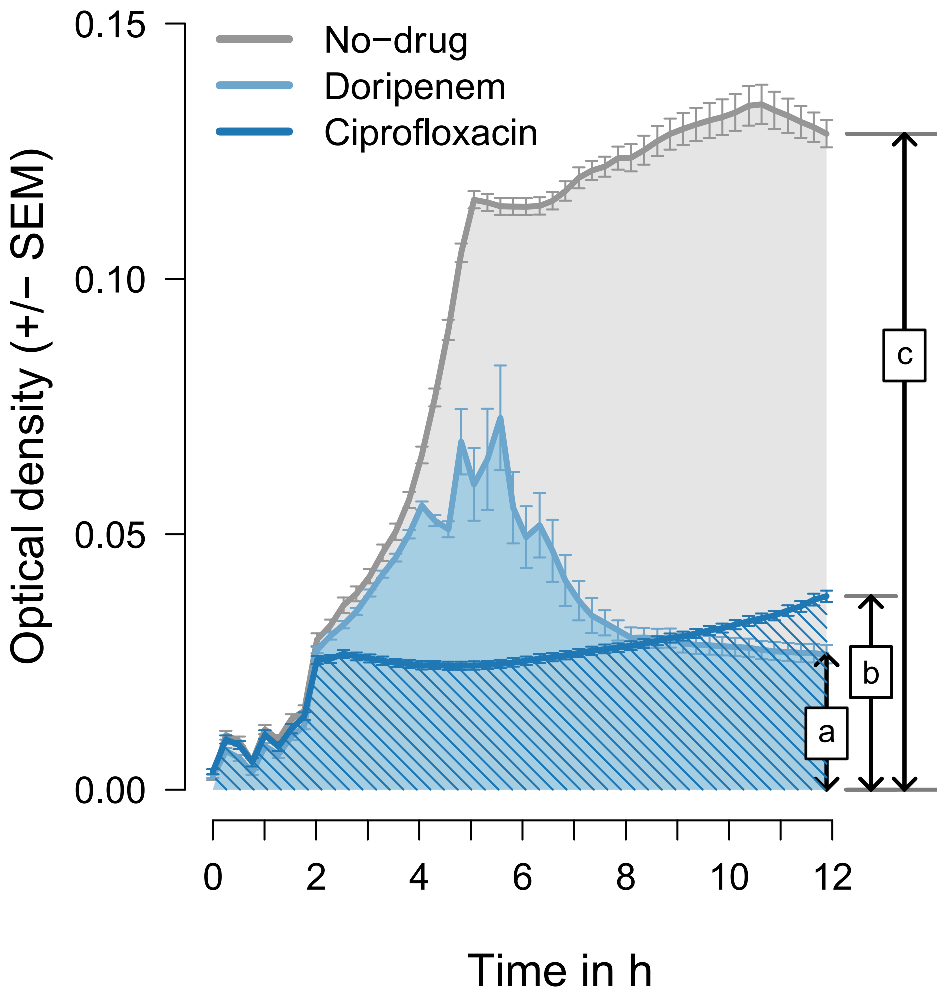

Figure S3. Time-growth curves of the first season in experiment 1.

Mean optical density for bacterial populations treated with doripenem (n = 40), ciprofloxacin (n = 40) or no-drug (n = 16) across time. The arrows a, b and c indicate bacterial density at the end of the season, *i.e.* after 12 h. We standardized drug concentrations so that the ratios of a/c and b/c were approximately 0.25. Error bars denote standard error of the mean (SEM). The mean area under the time-growth curve (AUC) is shaded according to drug treatment in season 1. AUC inhibition of an antibiotic A is defined as 1 – (AUC_A /$AUC_{no\text{-}drug}$) x 100%.

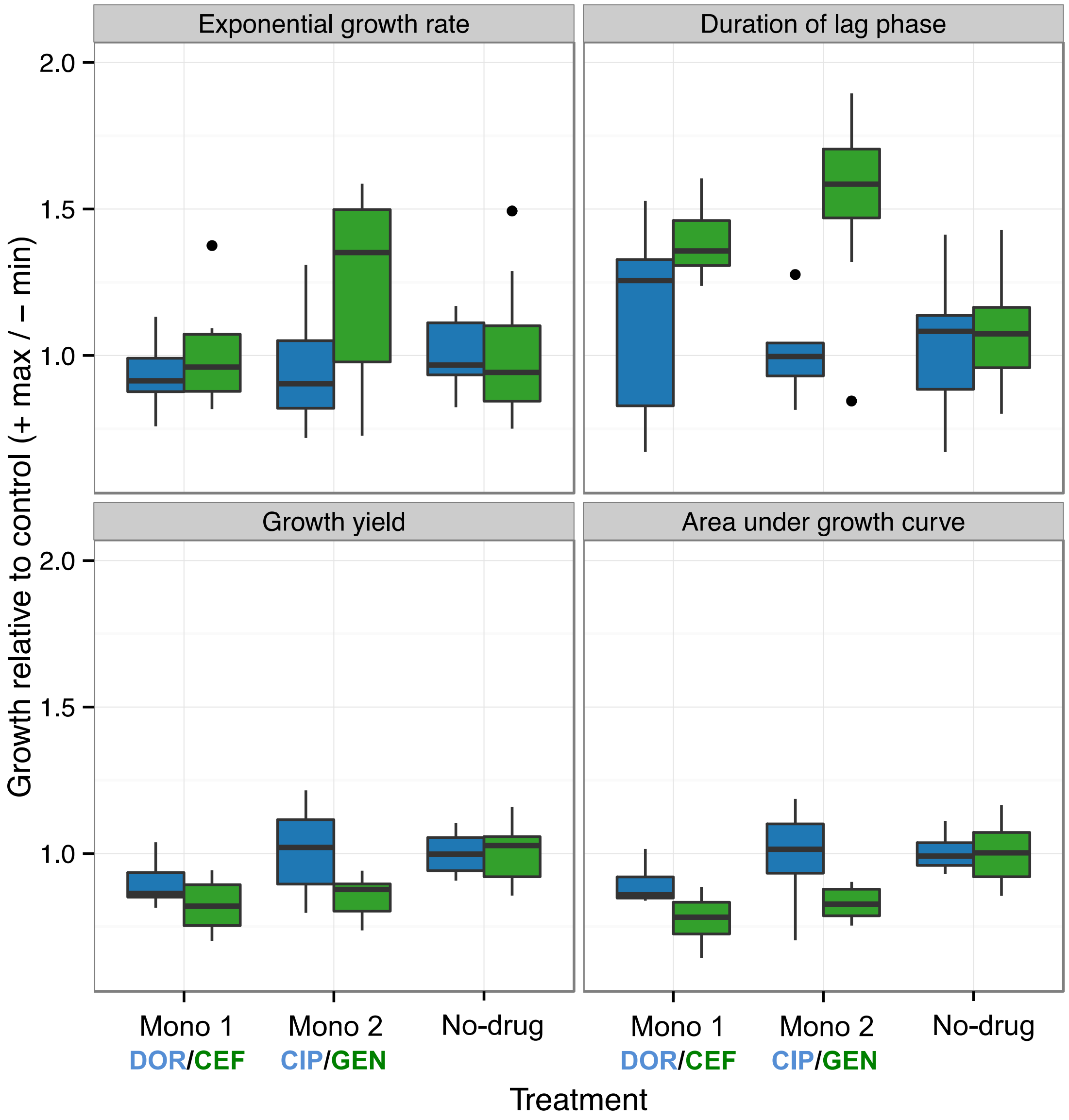

Figure S4. Fitness costs of evolving antibiotic resistance.

The boxplots show growth parameters for the treatments 1,2 and 11 obtained by fitting a logistic model to the growth curves (R-package 'grofit'; Kahm et al. 2010) in the antibiotic free season 17. We scaled the values by dividing through the mean of treatment 11 (no-drug control). The lines indicate the median value, the box spans 1st and 3rd quartile, whiskers show extreme values not larger than 1.5x interquartile range in both directions. Values outside the whiskers are plotted as points. We found no statistically significant differences in the costs of resistance within the antibiotic pairs.

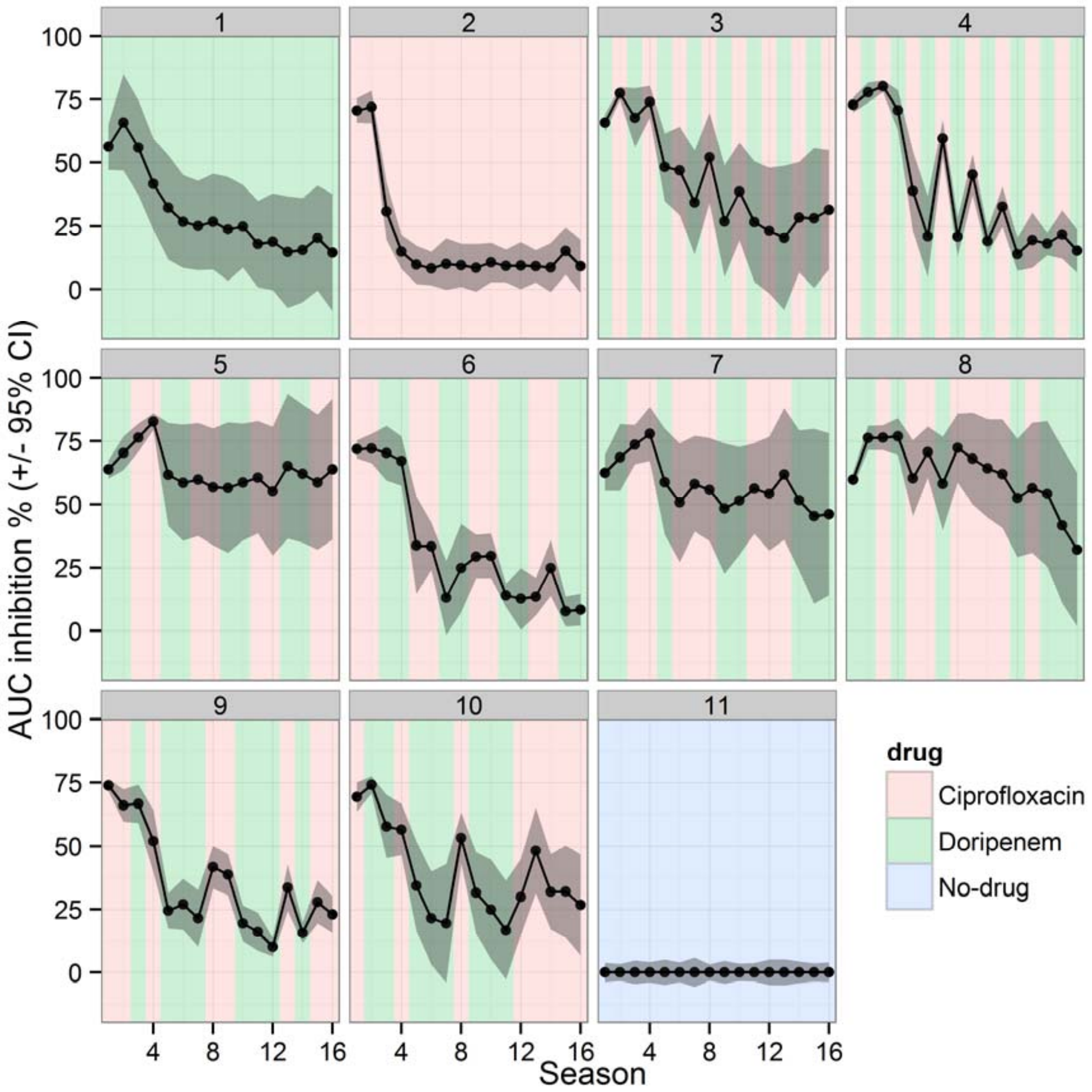

Figure S5. Evolutionary zig-zag dynamics for all treatments of experiment 1.

The lines show the means of eight replicate populations. The shaded ribbon represents the 95% confidence interval. The background shading depicts the antibiotic order.

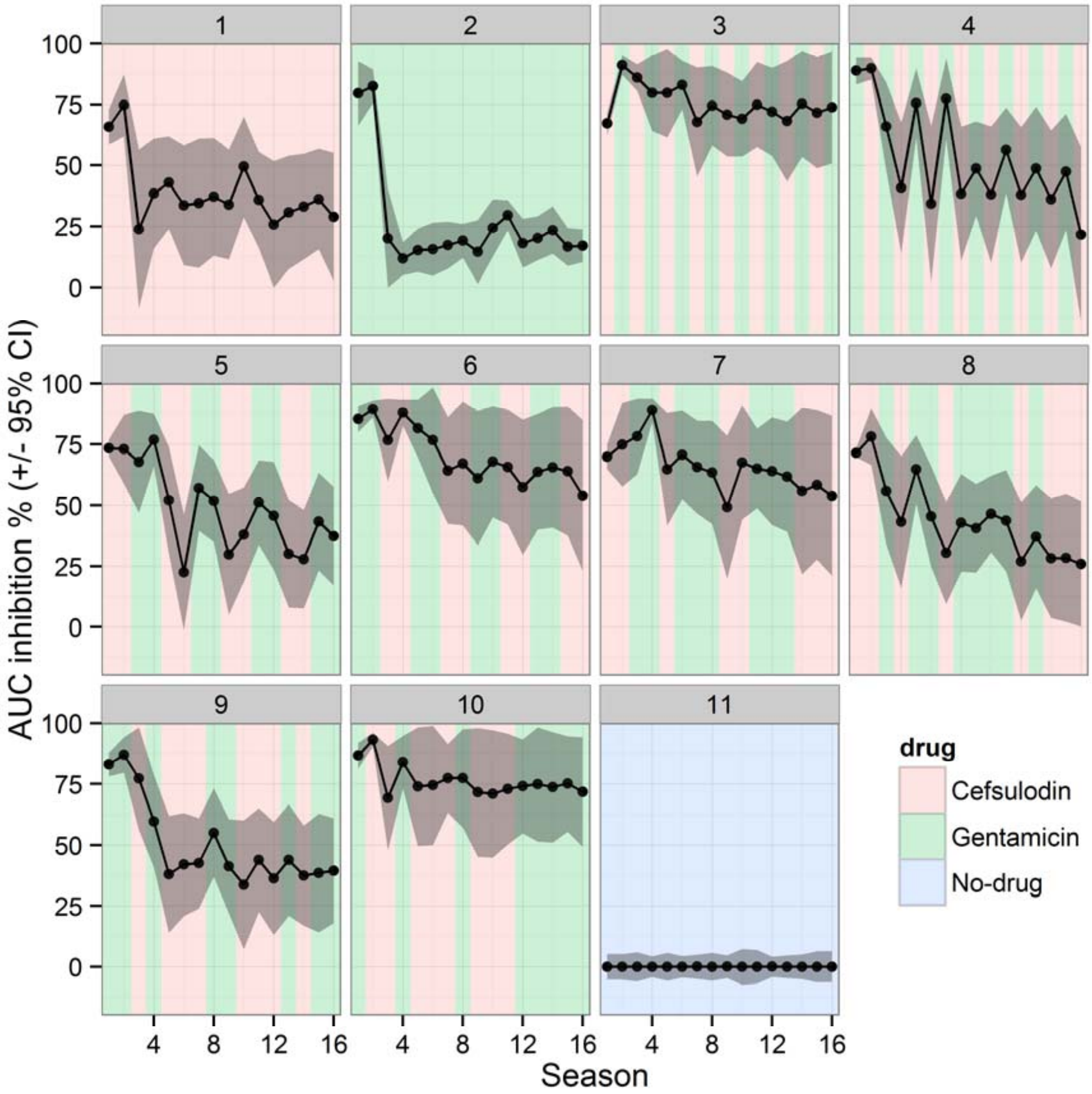

Figure S6. Evolutionary zig-zag dynamics for all treatments of experiment 2.

The lines show the means of eight replicate populations. The shaded ribbon represents the 95% confidence interval. The background shading depicts the antibiotic order.

Table S1. Significance of AUC inhibition across separate timescales for each antibiotic.

**Exp.**	**Antibiotic timescale**	**Comparison***	***z*-statistic¶**	***p*-value§**
1	Doripenem	Mono (1) *vs.* Regular (3-6)	0.123	0.902
1	Doripenem	Mono (1) *vs.* Random (3-6)	-0.182	0.902
1	Doripenem	Regular (3-6) *vs.* Random (7-10)	-0.482	0.902
1	Ciprofloxacin	Mono (2) *vs.* Regular (3-6)	-2.984	**0.004**
1	Ciprofloxacin	Mono (2) *vs.* Random (3-6)	-3.691	**< 0.001**
1	Ciprofloxacin	Regular (3-6) *vs.* Random (7-10)	-1.119	0.263
2	Cefsulodin	Mono (1) *vs.* Regular (3-6)	-1.153	0.436
2	Cefsulodin	Mono (1) *vs.* Random (3-6)	-1.056	0.436
2	Cefsulodin	Regular (3-6) *vs.* Random (7-10)	0.153	0.878
2	Gentamicin	Mono (2) *vs.* Regular (3-6)	-4.204	**< 0.001**
2	Gentamicin	Mono (2) *vs.* Random (3-6)	-3.620	**< 0.001**
2	Gentamicin	Regular (3-6) *vs.* Random (7-10)	-0.923	0.355

* The treatment protocols specified in the parentheses were grouped according to treatment type (Mono, Regular or Random; see methods for explanation of protocol numbers) and contrasted in the post hoc test.

¶ The *z*-statistic is obtained from the post-hoc test of the mixed linear model. In the model, AUC inhibition is the response variable, experimental seasons and treatment protocol are fixed factors and replicate populations are included as a nested random factor. The random effect of populations was statistically significant in all models (p <0.0001).

§ To account for multiple testing *p*-values were adjusted using the false discovery rate (fdr). Significant *p*-values are given in bold.

Table S2. Summary of statistics for the assessment of collateral sensitivity.

Experiment	Season	Antibiotic in evolution experiment	Antibiotic in test	*t*-statistic[¶]	*p*-value[§]
1	6	DOR	DOR	4.921	**<0.001**
1	6	DOR	CIP	0.652	0.580
1	7	CIP	DOR	2.449	**0.014**
1	7	CIP	CIP	-0.554	0.580
2	6	CEF	CEF	2.174	**0.030**
2	6	CEF	GEN	-2.115	0.069
2	7	GEN	CEF	2.305	**0.030**
2	7	GEN	GEN	-0.327	0.744

[¶] The *t*-statistic is obtained by comparison of the area under the dose response curve (AUC) of the tested populations (n = 3) with the AUC of ancestral populations assessed under exactly the same test conditions (n = 5), using a mixed linear model with clones nested in populations.

[§] *P*-values were adjusted by the false discovery rate (fdr). Significant values are given in bold.

Table S3. Summary of statistics for comparisons of cumulative OD.

		Experiment 1			Experiment 2		
**Comparison ***	**Tails¶**	***t*-statistic**	**df $**	***p*-value§**	***t*-statistic**	**df $**	***p*-value§**
Mono (1,2) *vs.* Alternation (3-10)	1	3.089	22.9	**0.008**	3.214	28.8	**0.010**
Regular (3-6) *vs.* Random (7-10)	2	0.722	56.1	0.606	-0.369	49.6	0.714
Alternations that start with β-lactam (3,5,7,8) *vs.* Alternations that start with non-β-lactam (4,6,9,10)	1	-3.916	37.7	**< 0.001**	0.378	52.9	0.714
Regular antibiotic switches at every season (3,4) *vs.* Regular antibiotic switches at every 2nd season (5,6)	2	0.676	25.4	0.606	-0.655	27.9	0.714
Treatment protocol 3 *vs.* Treatment protocol 4	2	-0.514	7.9	0.621	-1.791	12.6	0.195
Treatment protocol 5 *vs.* Treatment protocol 6	2	-2.423	7.0	0.092	2.833	12.7	**0.043**

***** The cumulative OD of replicate populations from the treatment protocols specified in the parentheses were grouped for the *t*-test. For details on treatment protocols see methods in main text.

¶ This column specifies whether the applied t-test was one-sided (1) or two-sided (2).

$ The number of degrees of freedom as estimated by the t.test() function in R.

§ *P*-values were adjusted by the false discovery rate to account for multiple comparisons (fdr).

Supporting references

Kahm, Matthias, Guido Hasenbrink, Hella Lichtenberg-Fraté, Jost Ludwig, and Maik Kschischo. 2010. 'Grofit: Fitting Biological Growth Curves with R'. *Journal of Statistical Software* 33 (7): 1–21.

Ritz, Christian, and Jens C. Streibig. 2005. 'Bioassay Analysis Using R'. *Journal of Statistical Software* 12 (5): 1–22.

Shannon, Claude Elwood. 1948. 'A Mathematical Theory of Communication'. *Bell System Technical Journal* 27: 379–423.
