# Supplementary figures and images for: Temporal variation in antibiotic environments slows down resistance evolution in pathogenic Pseudomonas aeruginosa
Source: Evol Appl. 2015 Oct 7;8(10):945–55. doi: 10.1111/eva.12330 (PMC4662347; doi:10.1111/eva.12330)

A

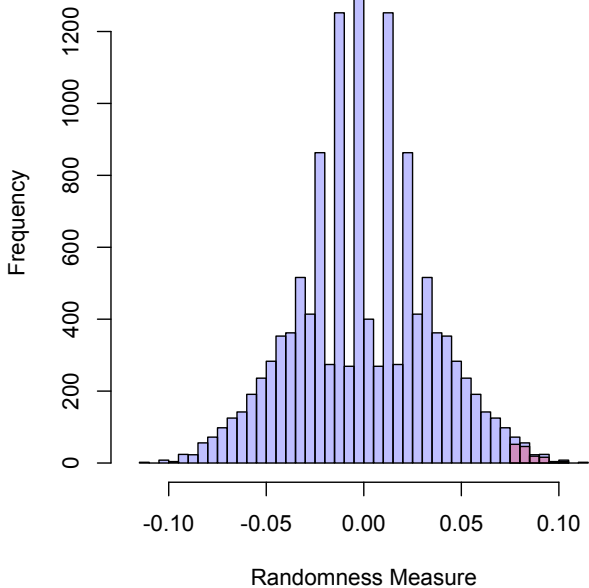

B

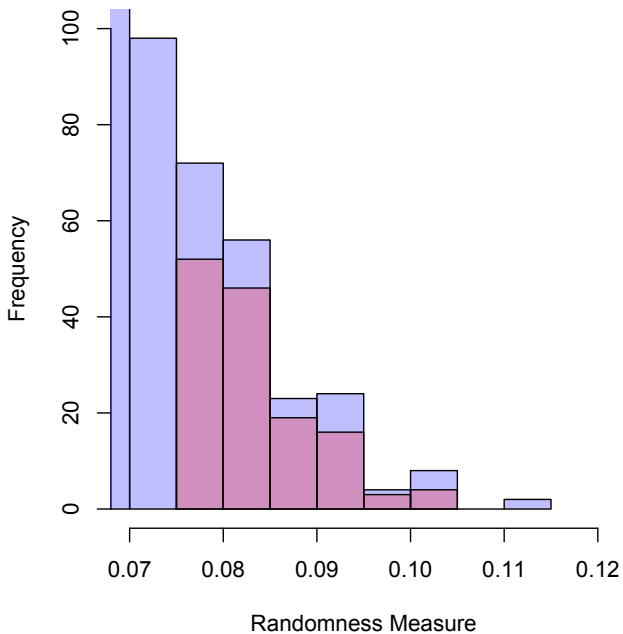

Supplement: Supplementary file 2 [file eva0008-0945-sd2.pdf]

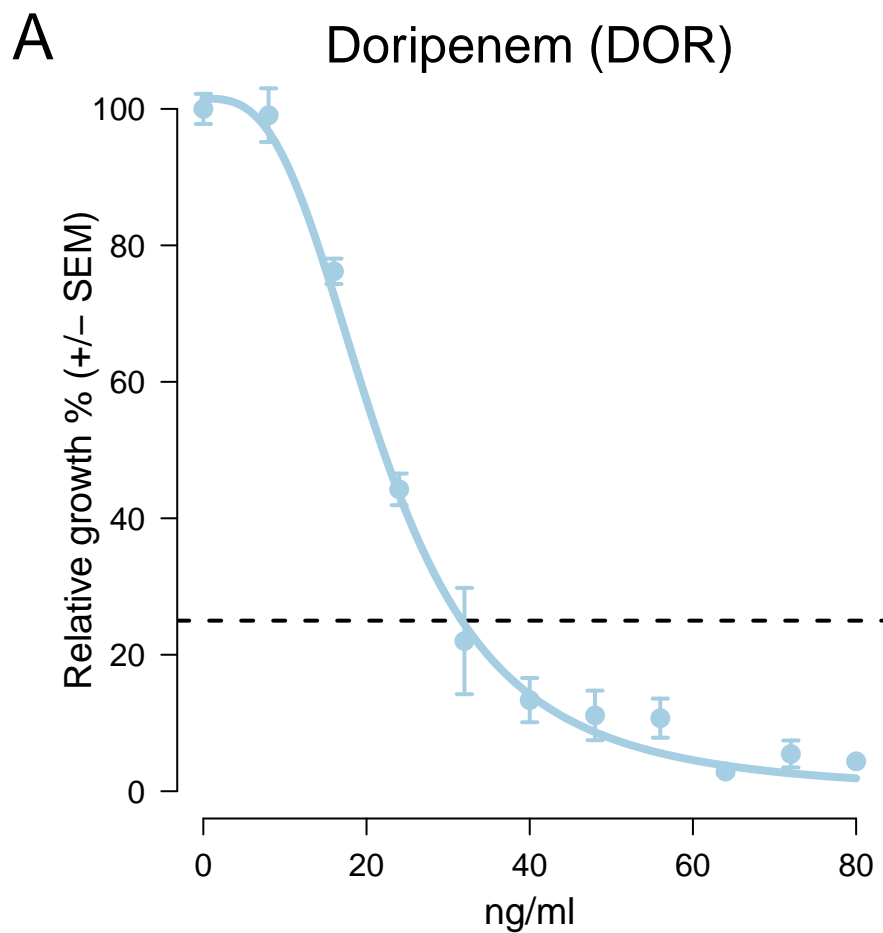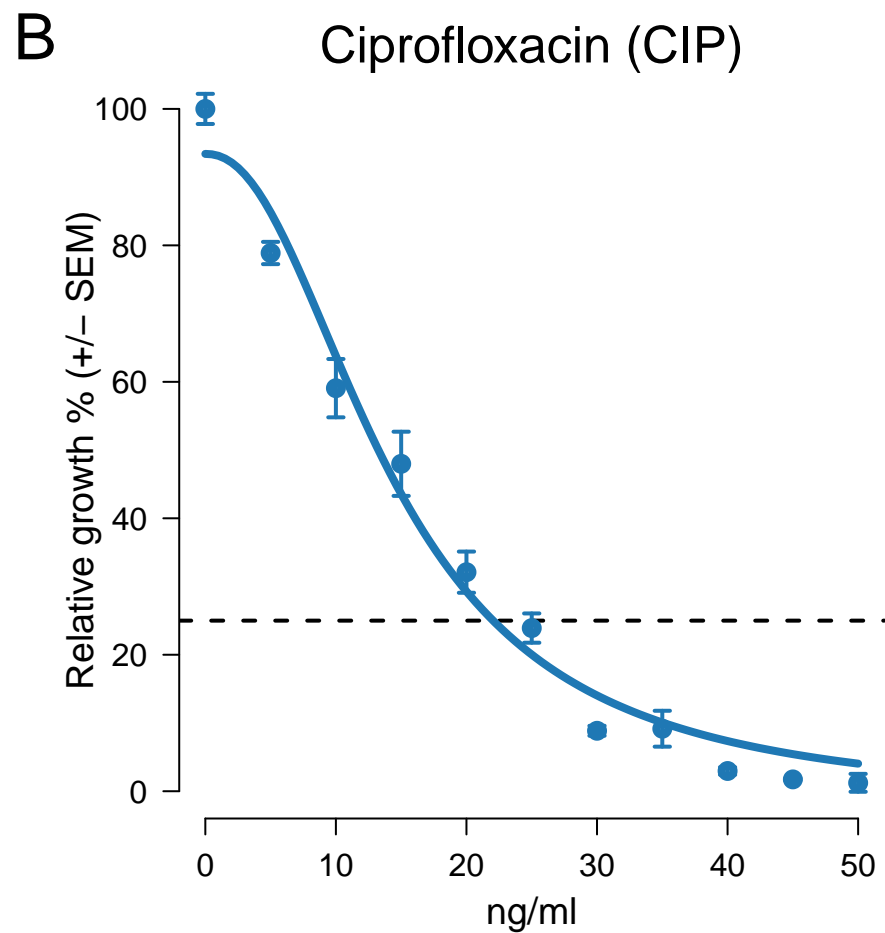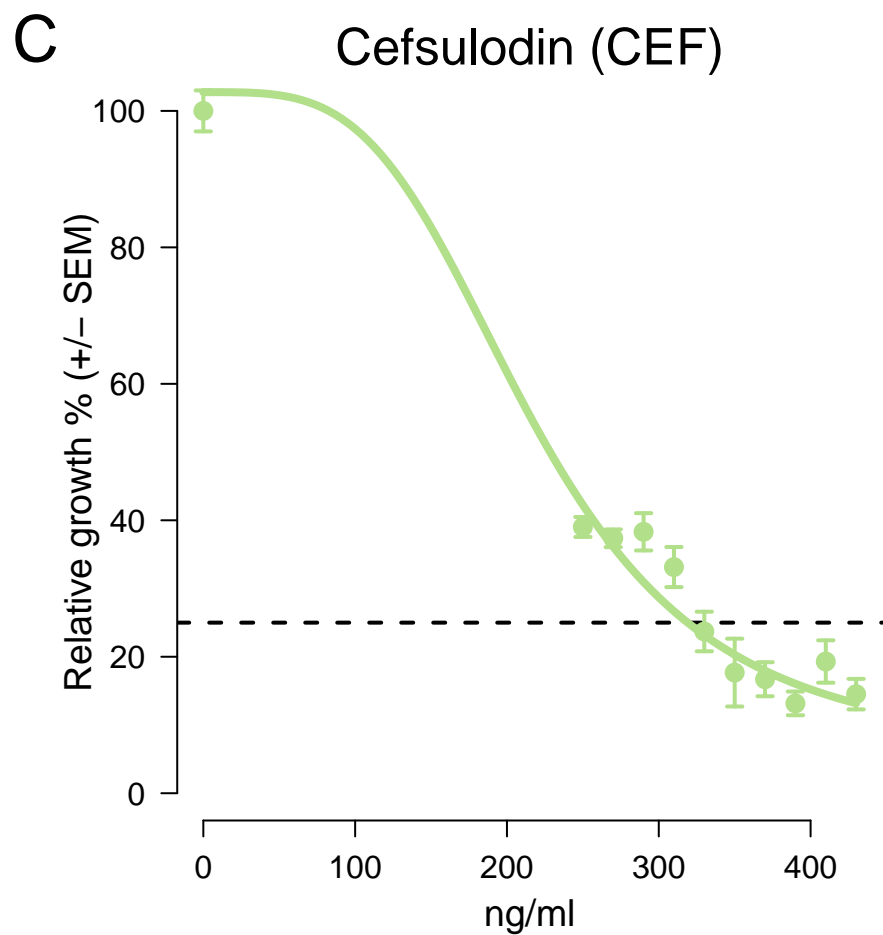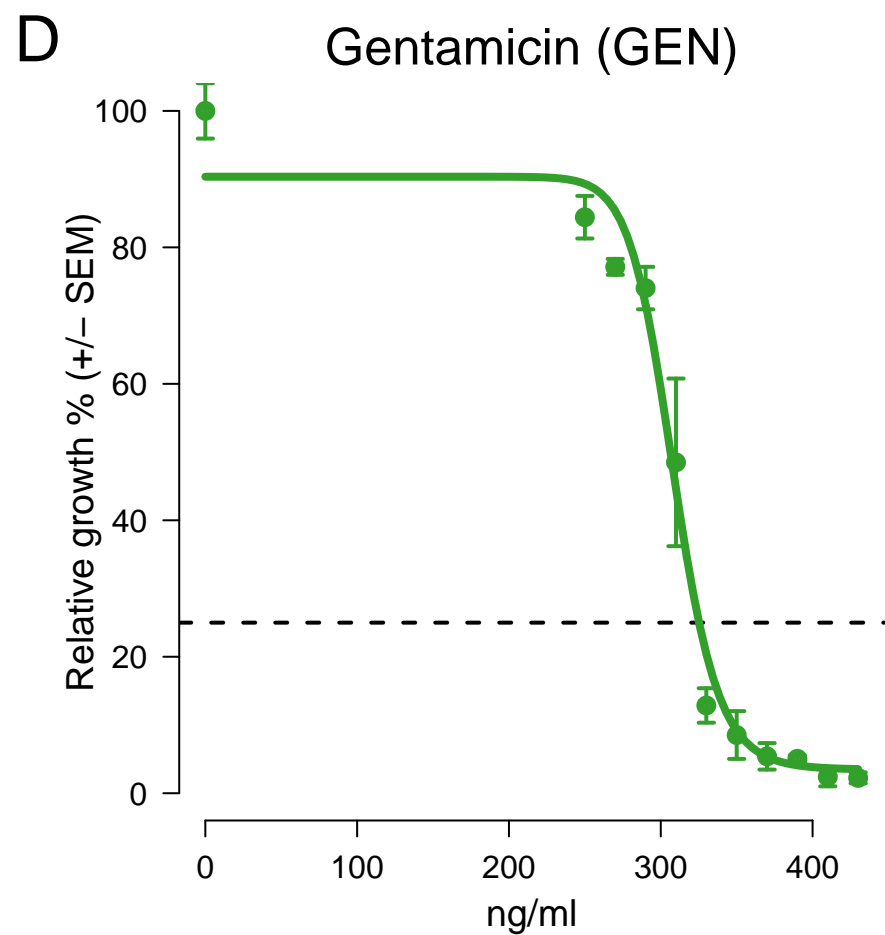

Supplement: Supplementary file 3 [file eva0008-0945-sd3.pdf]

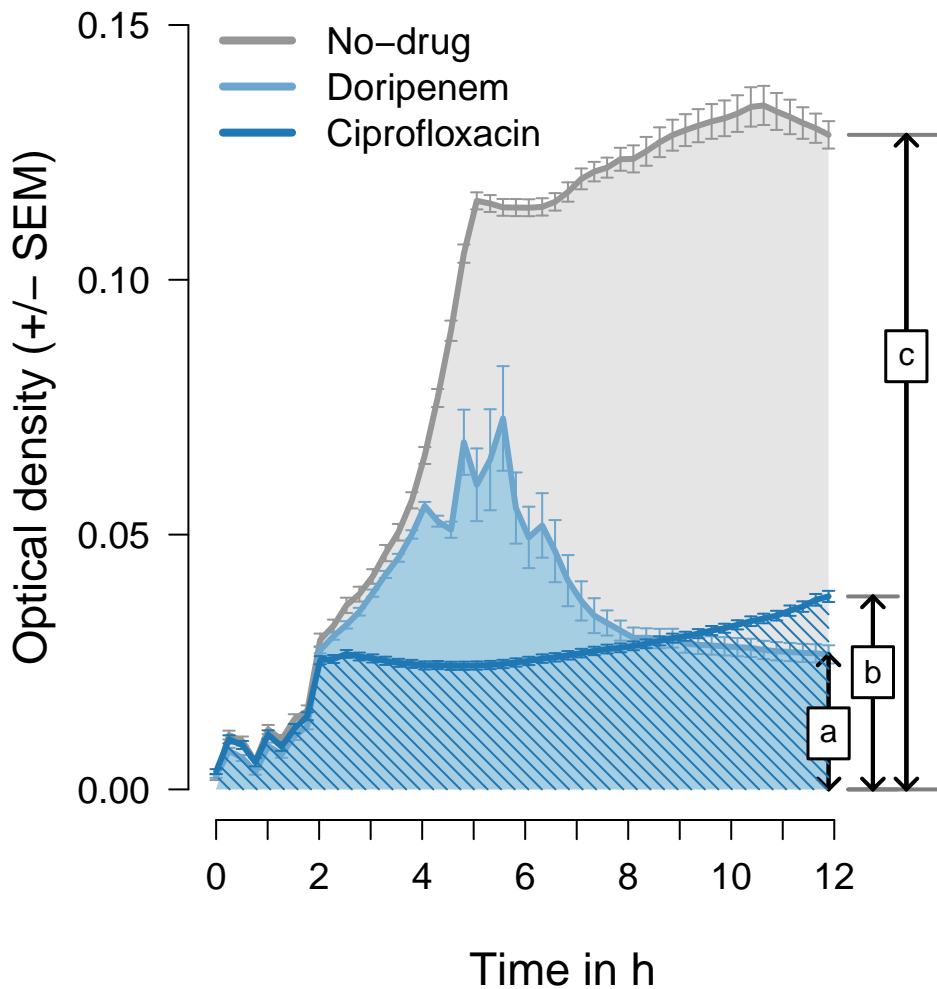

Supplement: Supplementary file 4 [file eva0008-0945-sd4.pdf]

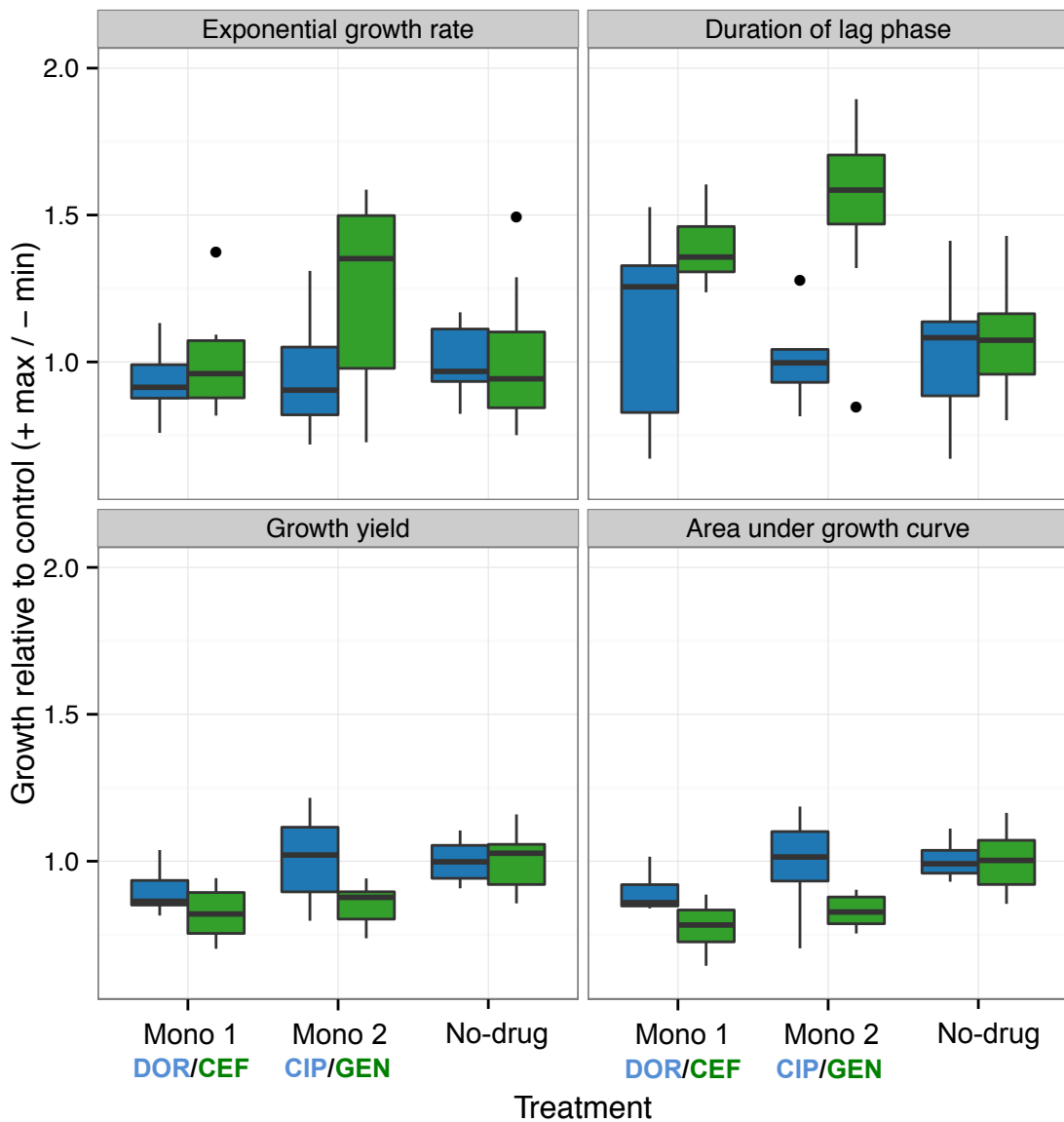

Supplement: Supplementary file 5 [file eva0008-0945-sd5.pdf]

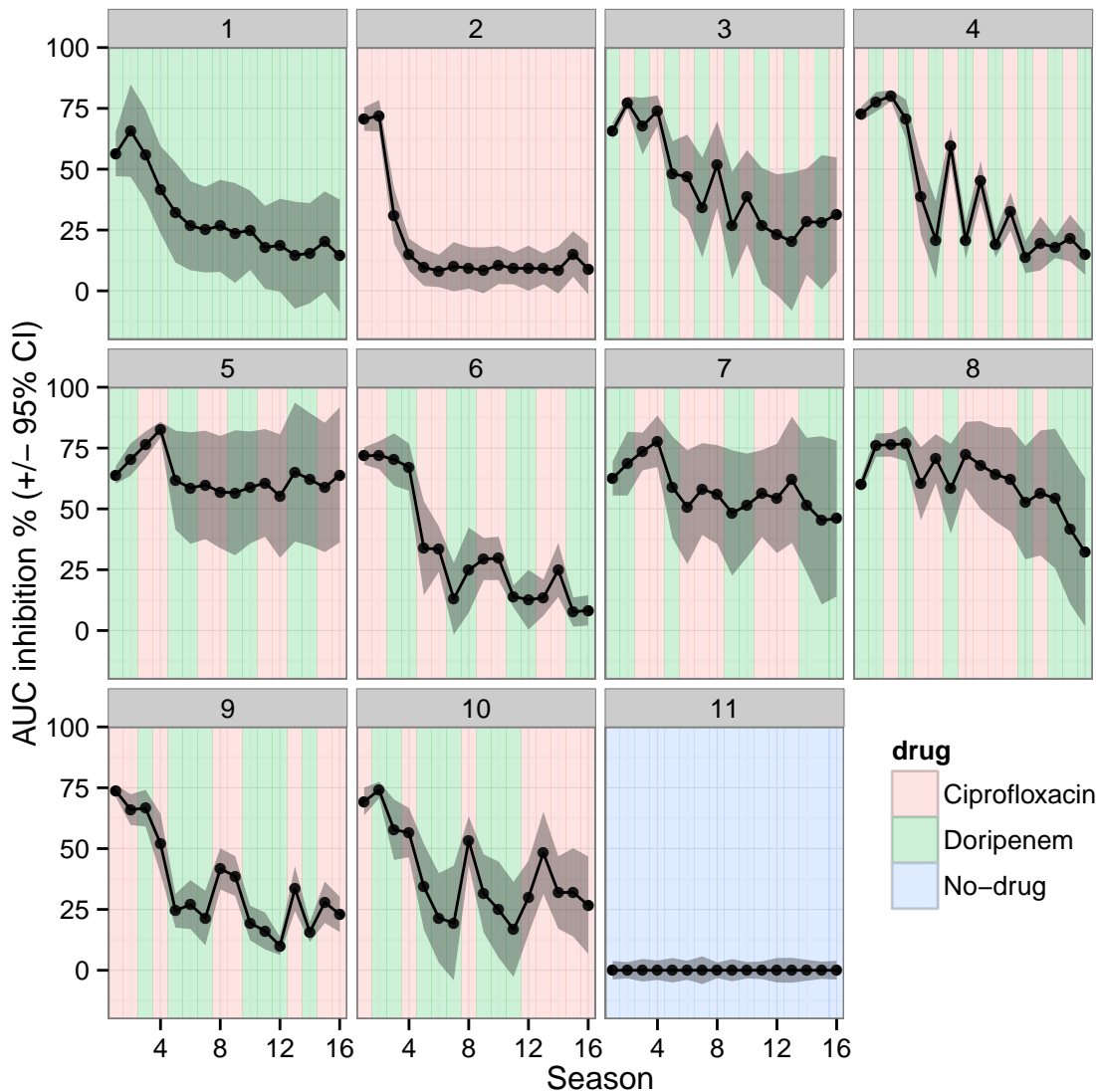

Supplement: Supplementary file 6 [file eva0008-0945-sd6.pdf]

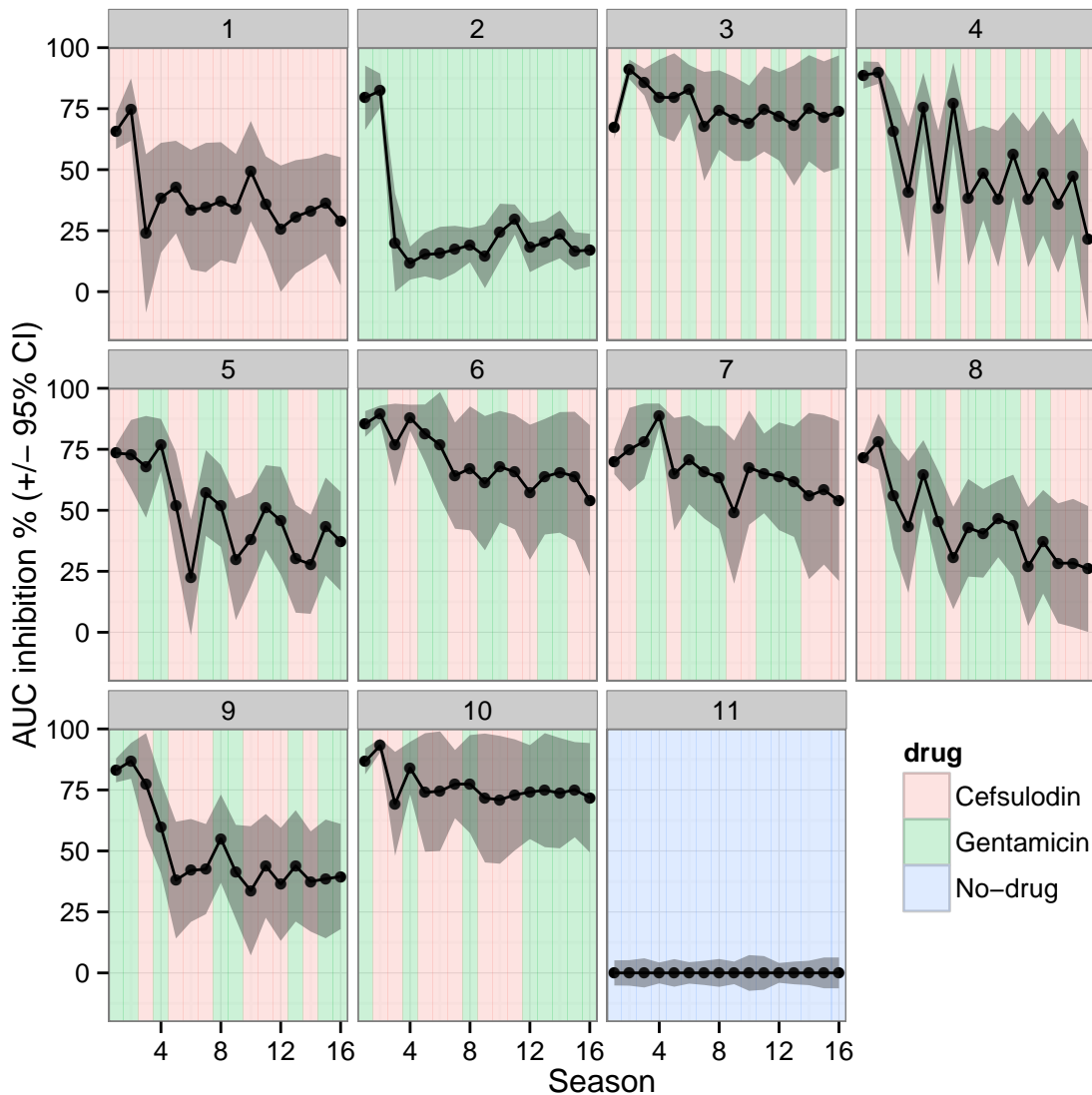

Supplement: Supplementary file 7 [file eva0008-0945-sd7.pdf]
